# Chromoanagenesis in the *asy1* meiotic mutant of *Arabidopsis*

**DOI:** 10.1093/g3journal/jkac185

**Published:** 2022-08-03

**Authors:** Weier Guo, Luca Comai, Isabelle M Henry

**Affiliations:** Genome Center and Dept. Plant Biology, University of California, Davis, Davis, CA 95616, USA; Genome Center and Dept. Plant Biology, University of California, Davis, Davis, CA 95616, USA; Genome Center and Dept. Plant Biology, University of California, Davis, Davis, CA 95616, USA

**Keywords:** chromothripsis, cancer, plant, meiosis, asy1, micronucleus, plant genetics and genomics

## Abstract

Chromoanagenesis is a catastrophic event that involves localized chromosomal shattering and reorganization. In this study, we report a case of chromoanagenesis resulting from defective meiosis in the MEIOTIC ASYNAPTIC MUTANT 1 (*asy1*) background in *Arabidopsis thaliana*. We provide a detailed characterization of the genomic structure of this individual with a severely shattered segment of chromosome 1. We identified 260 novel DNA junctions in the affected region, most of which affect gene sequence on 1 or both sides of the junction. Our results confirm that *asy1-*related defective meiosis is a potential trigger for chromoanagenesis. This is the first example of chromoanagenesis associated with female meiosis and indicates the potential for genome evolution during oogenesis.

**Plain Language Summary:**

Chromoanagenesis is a complex and catastrophic event that results in severely restructured chromosomes. It has been identified in cancer cells and in some plant samples, after specific triggering events. Here, we identified this kind of genome restructuring in a mutant that exhibits defective meiosis in the model plant system *Arabidopsis thaliana*.

## Introduction

Complex chromosomal rearrangements refer to genomic structure variation that involves at least 3 double-strand DNA breaks among 2 or more chromosomes (Pellestor *et al.* 2011). These changes can cause the truncation, relocation, or copy number variation (CNV) in multiple genes or gene regulatory elements, which can subsequently lead to dramatic phenotypic changes (Poot and Haaf 2015). Chromoanagenesis, caused by a single catastrophic genome restructuring event, and diagnosed by the presence of tens to hundreds of CNVs on a single chromosome, has been identified in many systems in the last decade (Liu *et al.* 2011; Stephens *et al.* 2011; Baca *et al.* 2013; Anand *et al.* 2014; Tan *et al.* 2015; Blanc-Mathieu *et al.* 2017; Cortés-Ciriano *et al.* 2020; Guo *et al.* 2021). It can be associated with multiple types of human cancer (Koltsova *et al.* 2019; Cortés-Ciriano *et al.* 2020), or with transgenic modifications used for plant genetic engineering (Tan *et al.* 2015; Liu *et al.* 2019; Fossi *et al.* 2019; Guo *et al.* 2021). The origin, mechanism, and potential effects of chromoanagenesis are just starting to be deciphered. Chromothripsis is a type of chromoanagenesis, characterized by the pulverization of a single chromosome and its random reassembly with limited copy number changes (Stephens *et al.* 2011; Korbel and Campbell 2013; Pellestor *et al.* 2021). Chromothripsis has been used to describe many extreme chromosome rearrangements in various systems (Carbonell-Bejerano *et al.* 2017; Liu *et al.* 2019; Mandáková *et al.* 2019). Besides chromothripsis, the 2 other types of processes included in chromoanagenesis—chromoanasynthesis and chromoplexy—can produce rearranged chromosomes as well but exhibit different features and result from different mechanisms (Liu *et al.* 2011; Baca *et al.* 2013). Here, in the absence of mechanistic information, we use the broader term chromoanagenesis to describe the chromosome restructuring patterns observed in our study.

MEIOTIC ASYNAPTIC MUTANT 1 (ASY1) is the *Arabidopsis* homolog of the yeast chromosome axis component HOP1. ASY1 plays an important role in meiotic recombination by regulating crossover assurance and interference (Caryl *et al.* 2000; Sanchez-Moran *et al.* 2008; Lambing *et al.* 2020). First observed in transgenic *Arabidopsis* mutants exhibiting reduced synapsis (Ross *et al.* 1997; Caryl *et al.* 2000), the presence of aneuploidy in the progeny of *ASY1* mutants suggests that the ASY1 mutation can also result in chromosome mis-segregation (Wei and Zhang 2010; Ferdous *et al.* 2012).

Here, we report a case of chromoanagenesis resulting from defective meiosis in the *asy1* mutant background in *Arabidopsis thaliana*. Specifically, a homozygous *asy1* mutant was crossed as a female to a wild-type male, and aneuploids were observed in the progeny (Pochon *et al.* 2022). Detailed characterization of the genome of one of these aneuploid individuals detected a severely shattered segment of chromosome 1, which was reminiscent of the consequences of chromoanagenesis. Our analyses identified 260 potential novel DNA junctions in this region, suggesting that defective *asy1* can trigger chromoanagenesis.

## Materials and methods

DNA from the *Arabidopsis* line exhibiting multiple CNVs was prepared for deep sequencing as follows. The genomic DNA was extracted from the leaf tissue and prepared for Illumina short-read sequencing as previously described (Henry *et al.* 2015). Demultiplexing and quality filtering was performed using a custom Python script (https://comailab.org/data-and-method/barcoded-data-preparation-tools-documentation/). Reads were mapped to the TAIR10 reference genome using BWA (Li and Durbin 2009). The output files (.sam files) were used for the subsequent analyses. Two controls were generated by pooling the low-sequencing read data from multiple wild-type *Arabidopsis* lines generated from a similar cross (Ler-1/Col-0 x Col-0) (Supplementary File 1) to obtain 2 control files of similar coverage as the target sample.

Dosage variation along nonoverlapping consecutive bins spanning the entire genome was documented as previously described (Henry *et al.* 2015; Tan *et al.* 2015). Bin coverage was normalized to the corresponding bin in a diploid control individual for normalization, by using a customized Python script (https://github.com/Comai-Lab/bin-by-sam). The expected relative read coverage of a diploid individual is expected to be close to 2, while values close to 1 and 3 represent deletion and duplication, respectively.

Novel DNA junctions were identified as described previously (Guo *et al.* 2021). Specifically, we searched for sequencing reads that span 2 genomic locations originally located at distant positions (>2,000 bp apart, or on different chromosomes) and that appear uniquely in the target *Arabidopsis* line but not in either of the 2 control samples. A custom Python script (https://github.com/guoweier/Poplar_Chromoanagenesis) was used to identify the potential genomic locations of the 2 breakpoints for each novel junction. Potential false positives were discarded based on a coverage threshold calculated as previously described (Guo *et al.* 2021). Next, PRICE assembly was applied to construct contigs spanning the novel DNA junctions (Ruby *et al.* 2013). These contigs were compared to the sequence of the *Arabidopsis* genome by BLAST (Camacho *et al.* 2009), with the expectation that the 2 sides of these novel DNA junctions should map to the expected regions specifically (no multiple mapping allowed).

To identify the origin of the shattered chromosome, single-nucleotide polymorphism (SNP) between Col-0 and L*er*-1 were collected as previously reported (Pochon *et al.* 2022). Next, we calculated the L*er*-1 allelic frequency along the genome of the shattered *Arabidopsis* line, following a method developed previously (Henry *et al.* 2015). Specifically, an mpileup file was created to record the allele and read coverage in each genomic position, followed with a simplification step to create a parsed-mpileup file, using a custom Python pipeline (http://comailab.genomecenter.ucdavis.edu/index.php/Mpileup) based on Samtools (Li *et al.* 2009). The parsed-mpileup file was used for calling SNPs between Col-0 and L*er*-1, and data from the selected SNPs were pooled into consecutive nonoverlapping bins. L*er*-1 allele frequency for each bin was calculated and visualized. Since the shattered *Arabidopsis* line was produced from a cross between a Col-0/L*er*-1 hybrid (female) and a WT-Col-0 (male), at least 50% Col-0 alleles were expected. The transmission of a L*er*-1 chromosome from the female parent results in 50% L*er*-1 alleles, while the transmission of a Col-0 chromosome results in 0% L*er*-1 alleles.

To analyze the genomic features surrounding the breakpoints, the frequency of gene space surrounding them was compared to the frequency of pseudo breakpoints randomly selected along the *Arabidopsis* genome. The annotation files of various genomic features of *A. thaliana* (TAIR10) was acquired from the GitHub repository (https://github.com/KorfLab/FRAG_project) associated with the breakpoint analysis previously performed on aneuploid *Arabidopsis* (Tan *et al.* 2015). Statistical analysis was performed as previously described (Guo *et al.* 2021).

## Results and discussion

**Fig. 1.**
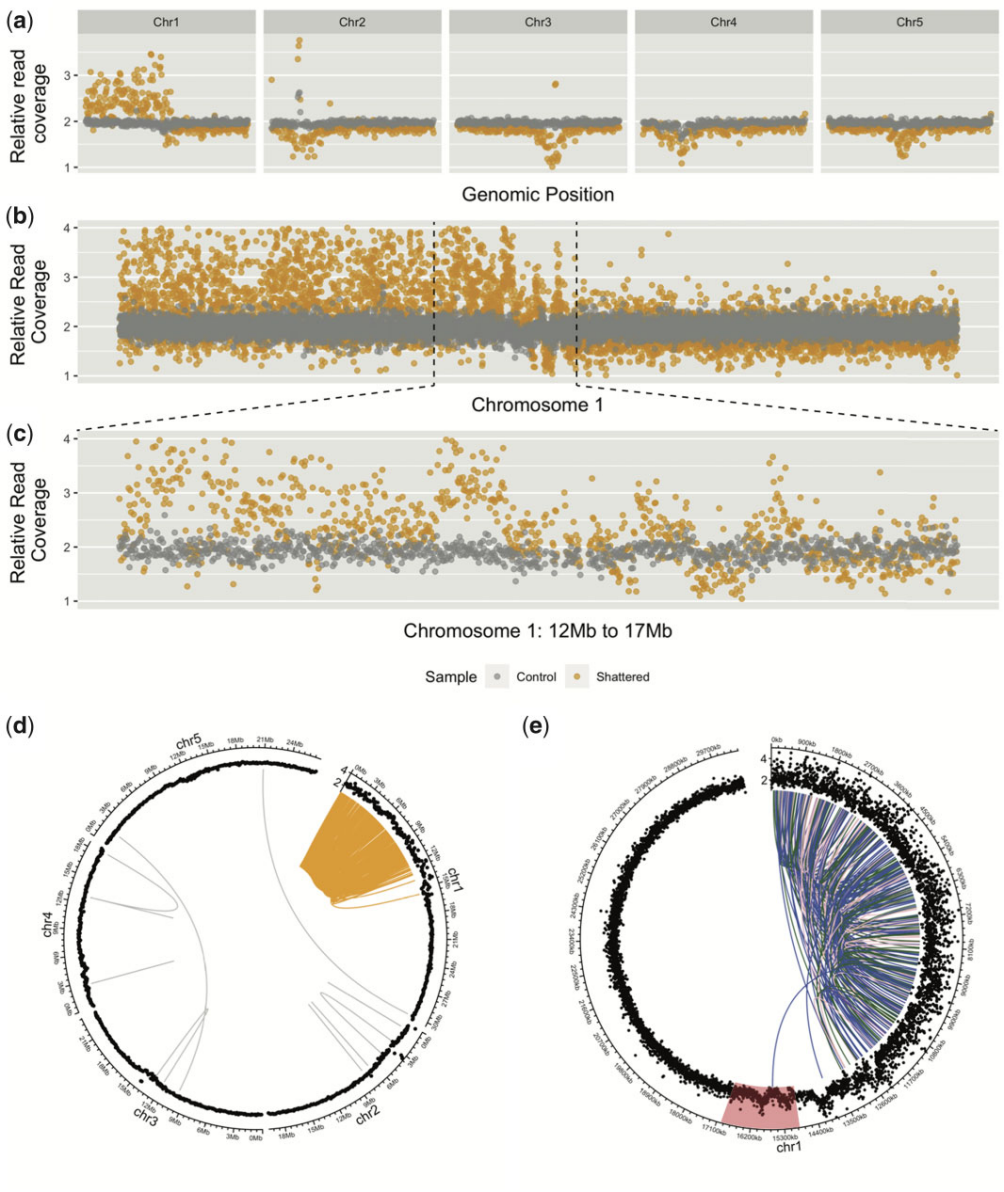
Characteristics of the genomic region with extreme dosage variations in the progeny of *asy1* mutant *A. thaliana*. a–c) Extremely dense CNV events on chromosome 1. Each dot represents the normalized read coverage in a bin along the genome. a) Relative read coverage across the whole genome (100-kb bins). b) Close-up of chromosome 1 (5-kb bins). c) Further close-up on the region of chromosome 1 that displays dense CNVs (5-kb bins). d, e) Breakpoints were highly enriched over the regions of chromosome 1 exhibiting clustered CNVs. The distribution of DNA junctions on all chromosomes of the *Arabidopsis* genome (d) and just chromosome 1 (e) are shown on a circos plots. The outermost layer indicates chromosomes. The next layer indicates relative read coverage, with 100-kb bins (d) and 5-kb bins (e). The center arcs represent the locations of the breakpoint pairs of the DNA junctions identified. d) The center arcs are colored in orange if both breakpoints are located within the CNV cluster, and in gray if both breakpoints fall elsewhere in the genome. e) The center arcs are colored in blue if the breakpoints are connected tail to head, in dark green if they are connected head to head, and in pink if they are connected tail to tail. The red highlighted region represents the chromosome 1 centromere. The CNV cluster represents the first 16.1 Mb of chromosome 1.

A recent study (Pochon *et al.* 2022) demonstrated that the genome of 1 offspring from a cross between a Col-0/L*er*-1 hybrid *asy1* mutants (asy1^*Col-0*^ × asy1^*L*er*-1*^, female) and a wild-type Col-0 (male) carries drastic genomic rearrangements. These rearrangements resemble the consequence of chromoanagenesis. Among the population of 176 individuals, a single line exhibited multiple CNVs, on chromosome 1, all clustered within the first half of the chromosome (from 1 to 16.1 Mb) (Fig. 1, a–c).

To confirm the occurrence of extreme chromosomal rearrangements in this individual, we searched for novel DNA junctions expected at the sites of chromosomal fragments reassembly. Specifically, we searched for Illumina sequencing reads that mapped to 2 distant genomic locations (>2,000 bp), indicating that 2 regions expected to be distant from each other in the reference genome are next to each other in the rearranged chromosome. We also expect that these novel DNA junctions are unique to the genome of this particular individual, and not present in its siblings. Based on these criteria, we identified 260 novel DNA junctions (Fig. 1, d and e and Supplementary File 2). For 95.7% (249 out of 260) of these junctions, both breakpoints fall within the shattered region on chromosome 1 (Fig. 1, e). The breakpoints of the remaining 11 junctions are both located elsewhere in the genome. This frequency of 1 breakpoint every 32 kb across the shattered region is much higher than previously observed following chromoanagenesis in other plant systems. Specifically, the frequency of breakpoints was 1/400 kb in chromoanagenetic individuals that originated from haploid induction crosses in *A. thaliana* (Tan *et al.* 2015) and 1 breakpoint per 250 kb for the chromoanagenesis events observed in the progeny of gamma-irradiated poplar pollen grains (Guo *et al.* 2021).

**Fig. 2.**
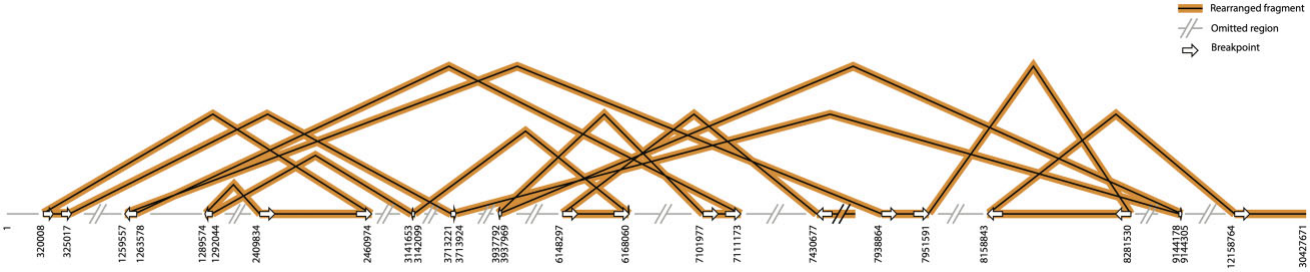
Potential structure of the rearranged chromosome. One of the restructured fragments resulting from the chromoanagenesis event, reconstructed based on the structure of the DNA junctions identified. The horizontal line represents chromosome 1. Segments involved in the rearrangement are shown in black, while segments not involved in this particular rearrangement are shown in gray. Each breakpoint is labeled with an arrow representing the joining orientation, and its original genomic positions.

Next, we attempted to reconstruct the structure of the novel chromosome based on the position and orientation of these breakpoints. In total, we were able to reconstruct 91 fragments from these 260 novel DNA junctions identified. The longest segment involved 13 novel DNA junctions (Fig. 2 and Supplementary File 3). The other reconstructed fragments are shorter, presumably because we did not identify all junctions in this sample, resulting in broken pieces in our reconstruction. Junctions that occurred within repeated regions, for example, are more likely to have been missed due to poor mapping specificity. Nevertheless, these results are consistent with the hypothesis that the shattered pieces reassembled randomly, in terms of orientation and order, resulting in a completely reorganized chromosome. This is consistent with the characteristics of chromothripsis (Korbel and Campbell 2013), and what we observed previously in poplar (Guo *et al.* 2021).

To characterize the properties of these novel DNA junctions, we investigated the DNA sequence context among breakpoint loci. Two window sizes, 1 and 10 kb, were used to calculate the enrichment ratio of gene space around the breakpoint loci. Statistical analysis suggested that breakpoint loci were significantly associated with gene-rich regions for both 1- and 10-kb bins (*P*-value <0.001) (Table 1). These results are consistent with previously documented chromoanagenesis events in plants, which also exhibited higher than expected frequency of breakpoints occurrence in genic regions (Tan *et al.* 2015; Guo *et al.* 2021). In addition to gene density, we characterized the potential enrichment of other genomic features, including chromatin states, transposable elements, or replication origins. The results suggest that breakpoint loci occurred more often in accessible chromatin regions, such as near transcription start sites, while they were significantly depleted in heterochromatin regions (Table 1) (Sequeira-Mendes *et al.* 2014).

**Table 1.** Enrichment ratio of novel breakpoints on other genomic features.

Genomic features		1kb window		10kb window		Description
		Enrichment ratio	*P*-Value	Enrichment ratio	*P*-Value	
Chromatin states	Chromatin state 1	1.58	1.59E−6^a^	1.31	7.27E−11^a^	TSS, promoter, 5′ UTR
	Chromatin state 2	1.28	0.026^b^	1.14	0.005^c^	Intergenic regions with proximal promoter elements
	Chromatin state 3	1.75	3.47E−8^a^	1.33	1.86E−9^a^	Transcription elongation signature
	Chromatin state 4	0.91	0.28	1.05	0.34	Intergenic regions with distal promoter elements
	Chromatin state 5	0.91	0.34	1.01	0.83	Polycomb-regulated chromatin, intergenic region
	Chromatin state 6	0.93	0.54	1.24	1.55E−4^a^	Gene bodies, intragenic region
	Chromatin state 7	1.23	0.09	1.3	2.68E−4^a^	Intragenic region, 55.6% coding sequence, 34.3% intros
	Chromatin state 8	0.38	4.00E−13^a^	0.49	3.43E−19^a^	AT-rich heterochromatin
	Chromatin state 9	0.04	4.91E−145^a^	0.11	8.47E−104^a^	GC-rich heterochromatin
Dnase I hypersensitive sites		1.36	3.24E−5^a^	1.21	3.15E−12^a^	
Gene		1.33	5 82E−21^a^	1.23	8.00E−30^a^	
mRNA		1.17	6.18E−9^a^	1.12	4.94E−12^a^	
Replication origin		1.5	0.047^b^	1.29	0.13	
Transposable element		0.31	3.59E−45^a^	0.42	2.44E−53^a^	

a *P*-value < 0.001.

b *P*-value < 0.0.5.

c *P*-value < 0.01.

Using in silico assembly of the junctions (see *Materials and Methods*), we were able to identify the exact location of the junctions and their exact sequences. Based on the specific sequence at these junctions, we determined that 50.4% (131 out of 260) of these junctions involved the joining of fragments in inverted configuration, while the other half involved 2 fragments coming together in the same orientation (Supplementary File 2). Junctions could be divided into 3 junction types: (1) microhomology is defined by the presence of an identical sequence (1–29 bp) on both sides of the junction, and resulting in a single repeat of the micro-homologous fragment at the resulting novel junction, instead of the expected 2 copies if the 2 fragments had come together directly; (2) perfect junctions involved the joining of the 2 ends with no modification at all, and (3) insertions involved the addition of a few base pairs (1–80 bp) between the 2 original DNA sequences. All 3 types were observed in this shattered line at the following rates: microhomology (63.8%), perfect joining (11.2%) and insertion (25%). Analysis of these precise locations demonstrated that 69.4% (361 out of 520) breakpoints occurred within a gene sequence. For 49.2% (128 out of 260) of the novel junctions, both breakpoints are located within coding regions (Supplementary File 4). This is expected to result in the loss of function of several genes and possibly in a few novel gene functions, in cases where the junction joined 2 different coding regions in phase.

Notably, the vast majority of the previously identified chromoanagenesis events in animals (Kloosterman *et al.* 2011; Zhang *et al.* 2015; Ly *et al.* 2019; Kneissig *et al.* 2019; Umbreit *et al.* 2020), and all of the characterized events in plants (Tan *et al.* 2015; Liu *et al.* 2019; Guo *et al.* 2021), have been associated with mitosis, usually during early embryo development (Tan *et al.* 2015; Papathanasiou *et al.* 2021), or male gametogenesis (Guo *et al.* 2021). Only a few studies have reported that chromoanagenesis can be correlated with meiotic divisions. Specifically, in human germ cells, chromoanagenesis has been demonstrated to occur during the meiotic divisions of spermatogenic cells and spermiogenesis (Kloosterman *et al.* 2011; Chiang *et al.* 2012). Extreme chromosomal rearrangements are also expected to occur following defects in female meiosis (Pellestor *et al.* 2014), but no case has been observed so far.

In this study, chromoanagenesis was detected in the offspring of an *asy1* homozygous mutant in a L*er*-1/Col-0 background. Specifically, the *asy1* allele in the L*er*-1 background was caused by a G nucleotide insertion caused by CRISPR-Cas9. The *asy1* allele in Col-0 corresponds to SALK_046272 T-DNA insertion line, near the ASY1 gene, which is located at 25,240,000 Mb on the lower arm of Chr1. Since both events occurred on chromosome 1, we cannot fully exclude the possibility that the T-DNA insertion played a role in the observed genomic instability. Nevertheless, it seems unlikely, based on the fact that the shattering and the T-DNA insertion are located on 2 different arms of chromosome 1, and the observation that none of the other progeny of this T-DNA insertion line exhibited chromoanagenesis.

The more likely explanation is that the ASY-1 mutation, which is known to affect crossover assurance and interference, resulted in altered recombination patterns and unbalanced chromosome segregation during meiosis (Lambing *et al.* 2020). Cytological evidence has shown the presence of unequal chromosome segregation during microsporogenesis in *asy1* mutants (Ross *et al.* 1997; Yuan *et al.* 2009; Wei and Zhang 2010; Cuacos *et al.* 2021). Cytological analysis of female sporogenesis in these plants also documented abnormal chromosome pairing and uneven chromosome segregation (Golubovskaya *et al.* 1992; Cao *et al.* 2021).

**Fig. 3.**
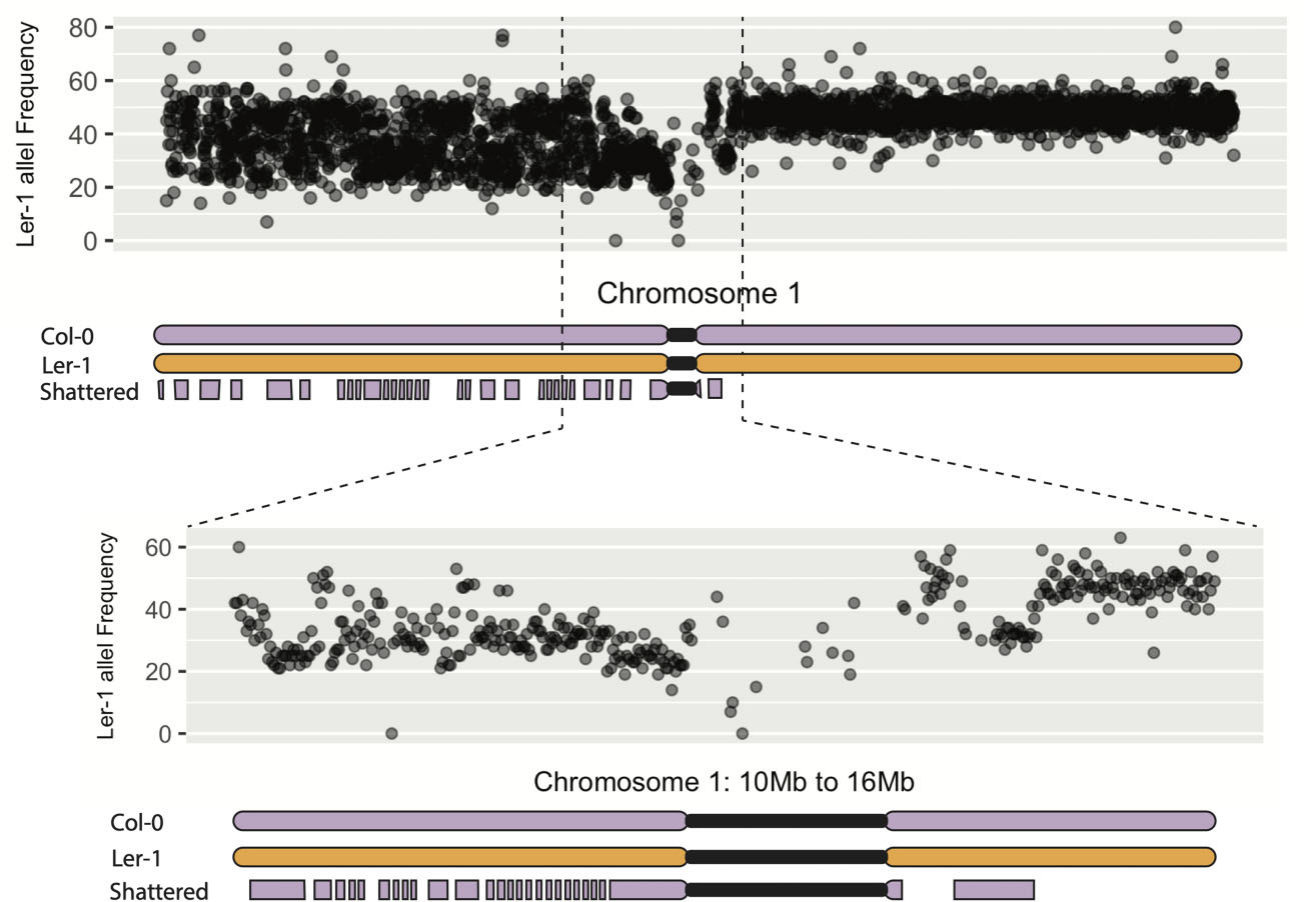
Parental allele variation in the shattered region. Variation in L*er*-1 allele frequency along chromosome 1 in the shattered *Arabidopsis* line. Each dot represents the percentage of L*er*-1 alleles within a 10-kb bin. A L*er*-1 allele frequency of 50% represents 1 copy of the L*er*-1 chromosome and 1 copy of the Col-0 chromosome. A frequency of 33% represents a 1:2 ratio of L*er*-1 and Col-0 alleles, with 1 chromosome copy from the Col-0 parent and 1 Col-0 and 1 L*er*-1 copy from the *asy1* mutant hybrid parent. The top graph represents L*er*-1 allele frequencies along the entire chromosome 1. The bottom graph represents a close-up of the pericentromeric region, from 10 to 16 Mb. The schematic drawing at the bottom of each graph represents the inferred karyotype of that region, with Col-0 chromosomes on the top and bottom (shattered) and the L*er*-1 chromosome in the middle. The fact that allelic percentages around the pericentromeric region switches between 50% and 33% indicate that 2 different haplotypes are inherited from the *asy1* mutant hybrid parent. This demonstrates that the shattered chromosome originated from mis-segregation during meiosis I (see Fig. 4).

**Fig. 4.**
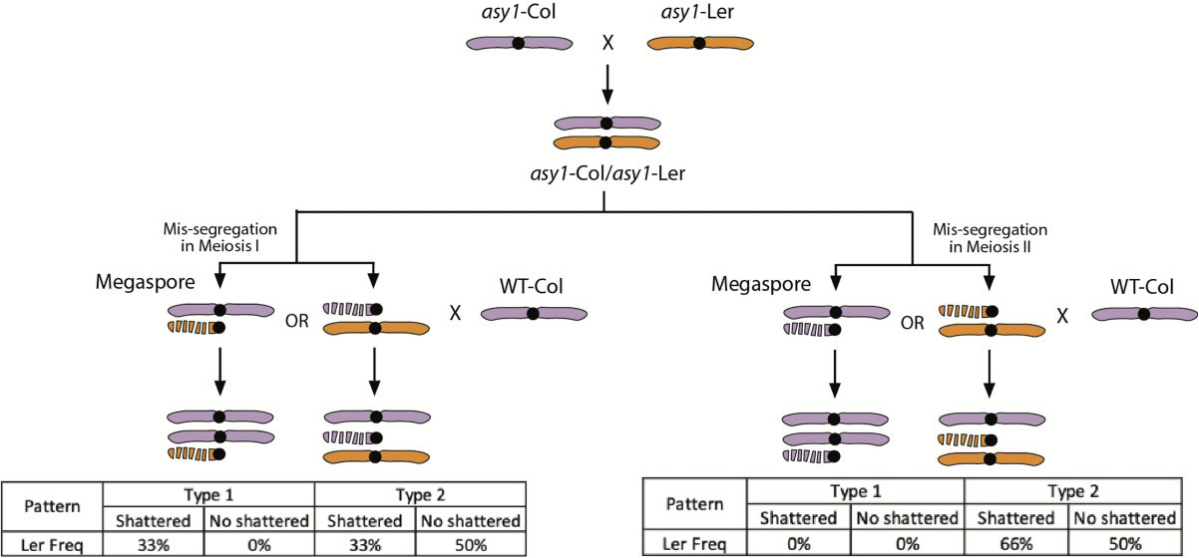
Schematic representation of the expected haplotype frequency for the *Arabidopsis* line with a shattered chromosome. The shattered *Arabidopsis* line is the product of 2 crosses. The first cross was performed between an *asy1*-Col-0 (female) and an *asy1*-L*er*-1 (male), producing *asy1*-Col-0/*asy1*-L*er-*1 hybrids. Next, the *asy1*-Col-0/*asy1*-L*er*-1 hybrids were used as females, and crossed to Col-0 (WT). During the second cross, the shattered chromosome resulted from mis-segregation during meiosis, either during meiosis I or meiosis II. Meiosis I missegregation causes the 2 homologous chromosomes to be inherited into the megaspore. In this case, the shattered chromosome is expected to carry a different haplotype from the intact chromosome. Meiosis II missegregation leads to the incorporation of 2 sister chromatids into the megaspore. In this case, the shattered chromosome is expected to carry the same haplotype as the intact chromosome. Considering the Col-0 genotype from the male parent, the final L*er*-1 genotype frequency in the shattered individual can inform us about the origin of the shattered chromosome. Meiosis I missegregation leads to 33% of L*er*-1 alleles along the shattered region, while meiosis II mis-segregation results in either 0% or 66% of L*er*-1 alleles. Recombination between L*er*-1 and Col-0 is not shown here but could lead to coinheritance of the recombinant chromatids, one of which would then undergo chromoanagenesis. Allele frequencies across the centromere and pericentromeric regions would remain the same as depicted here and are most indicative of the timing of mis-segregation.

To investigate at which stage of meiosis this missegregation occurred, we performed a haplotype analysis to examine the origin of the shattered chromosome. This analysis indicates that the percentage of L*er*-1 alleles oscillates between 33% and 50% within the shattered region. In contrast, it remains stably around 50% for the rest of chromosome 1 (Fig. 3). Furthermore, the presence of many regions exhibiting 33% L*er*-1 alleles indicates trisomy of the upper arm of chromosome 1. These data are consistent with the following scenario: 2 copies are intact, 1 Col-0 haplotype is from the Col-0 parent, and the other is a L*er-*1 haplotype from the hybrid mutant parent. The frequency of Ler-1 alleles over the shattered region goes back and forth between 33% and 50%. This indicates that the shattered chromosome carries the Col-0 haplotype, adding a copy of the Col-0 haplotype when it is present (33% L*er*-1). The regions that are missing from the shattered chromosome remain at 50% L*er*-1 from the 2 intact chromosomes. The fact that allelic frequencies oscillate between those 2 percentages (50% and 33%) throughout the shattered region suggests that the shattered chromosome was not the product of a recombination even in the mutant hybrid prior to mis-segregation. Finally, based on the expected percentage of parental alleles in various cases (Fig. 4), the observed percentages suggest that the shattered chromosome 1 originated from mis-segregation during meiosis I of megasporogenesis.

**Fig. 5.**
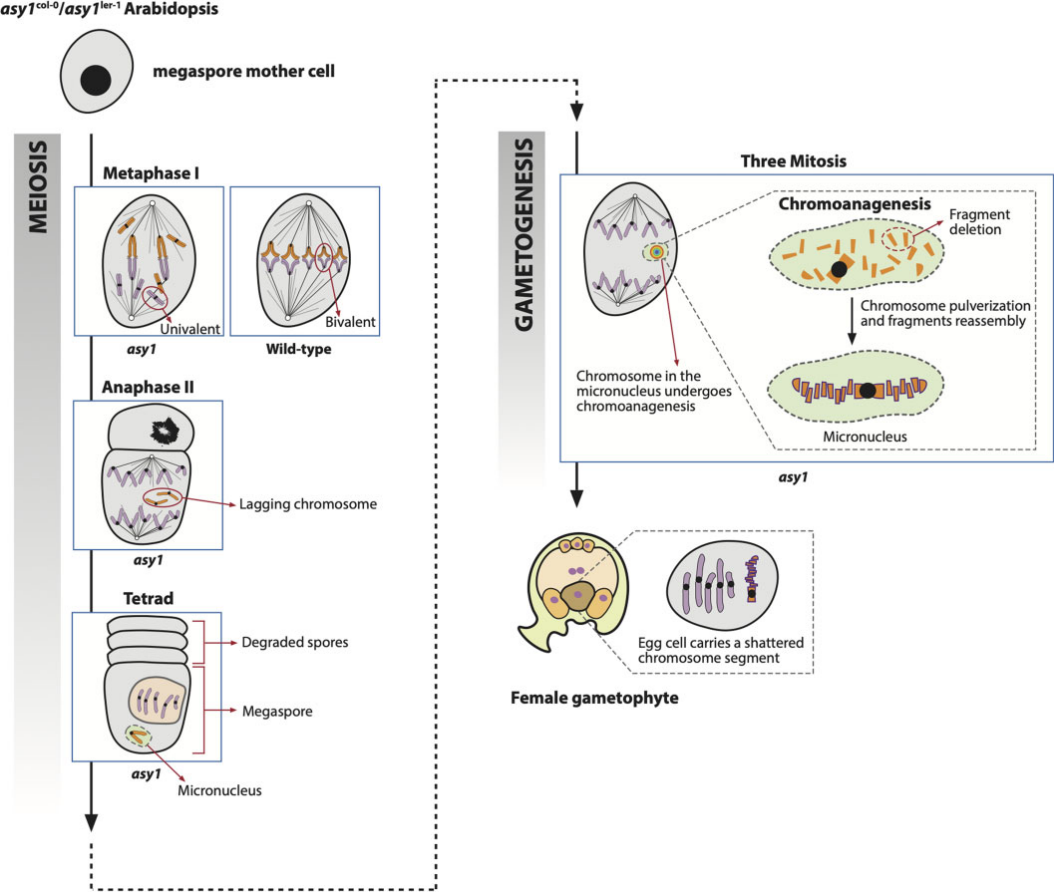
Proposed mechanism for chromoanagenesis in the *asy1* homozygous mutant. The megaspore mother cell from the *asy1* homozygous mutant exhibits chromosome mis-segregation during female meiosis. Specifically, the *asy1* mutation results in the formation of univalents at metaphase I, which leads to unbalanced chromosome segregation. During meiosis II, the missegregated chromosome lags and is incorporated into a micronucleus. In the following 3 mitosis during gametogenesis, the chromosome within the micronucleus is unable to synchronize with the mitotic division of the main nucleus and undergoes pulverization and restructuring, resulting in a chromosome with clustered structural variation. This shattered chromosome can be transmitted to the progeny if it is partitioned into the egg cell after micronucleus disassembly.

Micronuclei have been observed during male sporogenesis in *asy1* mutants with Col-0/L*er*-1 background (Pochon *et al.* 2022). Micronuclei have also been observed in haploid induction crosses, which also have generated chromoanagenetic events (Tan *et al.* 2015). It is thus possible that a similar suite of events are at play here. Specifically, chromosome mis-segregation occurred during female sporogenesis, resulting in the formation of a micronucleus carrying a chromosome laggard. Fragmentation and reorganization of the chromosome entrapped within the micronucleus subsequently created the shattered chromosome (Fig. 5). Together, our results provide the first example of chromoanagenesis triggered during female meiosis.

The fact that this *Arabidopsis* line produced a viable plant despite carrying an extremely shattered chromosome may be explained by the fact that the rearranged segments are present in 3 copies in a diploid background. Therefore, any negative functional effect of the shattering and rearrangements, including to protein sequences or reduced gene expression, are buffered by the presence of 2 intact copies of chromosome 1. The same situation applies to the *Arabidopsis* lines that underwent chromoanagenesis from haploid induction crosses: the rearranged chromosome or chromosomal segments were present as an extra copy of a trisomy (Tan *et al.* 2015).

Our result further suggests that *A. thaliana* is able to tolerate this extreme restructuring process during meiosis and survive through fertilization and embryogenesis. Unfortunately, seed was not collected from this particular line, so it is unclear if the shattered chromosome was transmissible sexually. Sexual transmission of a similar shattered chromosome was reported in a previous study in *Arabidopsis* though (Tan *et al.* 2015).

Chromoanagenesis-like rearrangements have been previously reported as potentially associated with a role in shaping the genome of camelina and the genus Cucumis (Mandáková *et al.* 2019; Zhao *et al.* 2021). Plant species such as *A. thaliana*, with powerful genetic resources could become a valuable system for investigating the mechanisms underlying extreme chromosomal rearrangement and eventually unraveling the pathways leading to chromoanagenesis, and their potential role in plant genome evolution.

## Conclusions

We describe a case of chromoanagenesis that is remarkable by the high frequency of new DNA junctions produced and because it results from asynapsis during female meiosis. The event demonstrates the potential for karyotypic innovation in connection to oogenesis.

## Supplementary Material

jkac185_Supplementary_Data

## Data Availability

The sequences reported in this paper have been deposited in the National Center for Biotechnology Information BioProject database (BioProject ID: PRJNA723952). Supplemental material is available at *G3* online.
