# Supplementary material for: Chromoanagenesis in the asy1 meiotic mutant of Arabidopsis
Source: G3 (Bethesda). 2022 Aug 3;13(2):jkac185. doi: 10.1093/g3journal/jkac185 (PMC9911071; doi:10.1093/g3journal/jkac185)
Supplement: jkac185_Supplementary_Data [file jkac185_supplementary_data.zip › jkac185_Supplementary_Data/Supplemental_Material_Legends_G3-2022-403647.docx]

**Supplementary Files**

[**Supplementary File 1**](https://docs.google.com/spreadsheets/d/1Kj_mHlzzK3c9hkI55v_axzI0cXdcLDA3b2K3xs15lbs/edit#gid=0)**. List of the wild-type Arabidopsis lines used for generating two controls.**

[**Supplementary File 2**](https://docs.google.com/spreadsheets/d/1cFnigJ1fVCQQYtnGc0zmHoahjcDwEaRY/edit#gid=217020782)**. Summary of all novel DNA junctions.**

[**Supplementary File 3**](https://docs.google.com/spreadsheets/d/1O0VQACxE-n92cLgdRtrqmjkqkpMFslDS/edit#gid=1230014644)**. List of rearranged fragments from novel DNA junctions.**

[**Supplementary File 4**](https://docs.google.com/spreadsheets/d/1Bef5VPAUE_YBHsJip_GymAjgRSuLpZdN/edit#gid=347509422)**. Breakpoints inside gene sequence.**
